# Mass Development of Diazotrophic Cyanobacteria (*Nostocales*) and Production of Neurotoxic Anatoxin-a in a *Planktothrix* (*Oscillatoriales*) Dominated Temperate Lake

**DOI:** 10.1007/s11270-016-3004-y

**Published:** 2016-08-10

**Authors:** Magdalena Toporowska, Barbara Pawlik-Skowrońska, Renata Kalinowska

**Affiliations:** 1 Department of Hydrobiology, University of Life Sciences in Lublin, Akademicka 13, 20-950 Lublin, Poland; 2 Centre for Ecological Research, P.A.S., Experimental Station, Niecała 18, 20-080 Lublin, Poland

**Keywords:** Anatoxin-a, DIN/DIP ratio, *Anabaena*, *Aphanizomenon issatschenkoi*, Nutrients, Hypertrophic lake

## Abstract

In spite of extensive studies on multispecies toxigenic cyanobacterial blooms, they are still difficult to eliminate, and factors regulating their succession and toxin production remain still to discover. A 4-year study revealed periodical mass development of diazotrophic *Nostocales* such as *Dolichospermum* spp. (previously *Anabaena*), *Aphanizomenon gracile* and expansive *Cuspidothrix* (previously *Aphanizomenon*) *issatschenkoi* in a lake affected by perennial blooms of *Planktothrix agardhii* (*Oscillatoriales*). Compared to *Oscillatoriales*, *Nostocales* reached the highest total biomass (up to 16 mg L^−1^) and contributed nearly 33–85 % to the total biomass of filamentous cyanobacteria at higher water temperatures (average values 17.5–22.6 °C) and higher ratio (11.8–14.1) of dissolved inorganic nitrogen to dissolved inorganic phosphorus (DIN/DIP). Species structure of *Nostocales* changed considerably from year to year as indicated by the Jaccard similarity index (0.33–0.78). Concentrations of intracellular anatoxin-a (ANTX) ranged from 0.03 to 2.19 μg L^−1^ of the lake water, whilst extracellular toxin reached up to 0.55 μg L^−1^. The highest positive correlations were found between the intracellular ANTX and the biomass of *Dolichospermum* spp. (*R*^2^ = 0.73) and *C. issatschenkoi* (*R*^2^ = 0.43–0.65). Our study suggests that ANTX production by *Dolichospermum* depended mainly on water temperature, whereas that by *C. issatschenkoi* was related to water conductivity and DIN/DIP ratio. P-PO_4_ concentrations also seemed to be important. The relatively short-term mass development of neurotoxic *Nostocales* is an additional threat to shallow, highly eutrophic water bodies continuously affected by *Oscillatoriales* blooms and may be controlled mainly by the DIN/DIP ratio. ANTX should be considered as a pollutant of freshwaters.

## Introduction

Water blooms caused by toxin-producing cyanobacteria are a serious problem not only in many eutrophic but also in mesotrophic and oligotrophic water bodies in the world (Henriksen et al. 1997; Lepistö et al. 2005; O’Neil et al. 2012; Kobos et al. 2013; Pearl 2014; Fernández et al. 2015). Numerous factors such as water temperature (preferred temperature >15 °C), light attenuation, vertical water mixing and turbidity, flushing rates, residence time, nutrient levels and ratios affect determination of cyanobacterial assemblage composition (i.e. N_2_-fixing vs. non-fixing taxa) and biomass (Rapala et al. 1993; Dokulil and Teubner 2000; Pearl 2008; O’Neil et al. 2012). In spite of extensive studies and present knowledge on cyanobacteria ecology (Dokulil and Teubner 2000; Rapala et al. 1993; Pearl 2008; O’Neil et al. 2012; Molot et al. 2014; Fernández et al. 2015), prediction, prevention and successful elimination of cyanobacterial blooms are still difficult or sometimes even impossible (Molot et al. 2014; Cirés and Ballot 2016). Cyanobacterial blooms affect aquatic ecosystems. For example, they influence physicochemical features of water by increasing the pH and redox potential of water, decreasing light conditions and so on (Oliver and Ganf 2000). As a consequence, disappearance of submerged macrophytes and simplification of aquatic biocenoses occur (Scheffer 1998). Toxin production by bloom-forming cyanobacteria is an additional threat for living organisms including humans (Welker and Döhren 2006; Van Apeldoorn et al. 2007; Testai et al. 2016).

Recently, more attention has been paid to anatoxin-a (ANTX) produced by some species of freshwater cyanobacteria. This potent nicotinic agonist has caused animal fatalities around the world (Briand et al. 2003; Puschner et al. 2008). Potential ANTX producers including *Dolichospermum*, *Aphanizomenon*, *Raphidiopsis*, *Cylindrospermum* and others (Sivonen et al. 1990; Rapala et al. 1993; Bumke-Vogt et al. 1999; Ballot et al. 2010) may be easily overlooked due to their relatively short-term mass occurrence. As suggested by O’Neil et al. (2012), climate warming may support more frequent mass development and longer duration of blooms of ANTX producers, as well as expansion of invasive species (Cirés and Ballot 2016) such as *Cylindrospermopsis raciborskii* and *Cuspidothrix issatschenkoi*. ANTX is harmful to aquatic fauna (e.g. planktonic invertebrates, benthic insect larvae and ichthyofauna; Claska and Gilbert 1998; Oberemm et al. 1999; Toporowska et al. 2014) and plants (Mitrovic et al. 2004). Moreover, ANTX accumulation has been recently found in muscles of freshwater fish roach, Prussian carp, perch and bream being an ingredient in human diet in Europe (Pawlik-Skowrońska et al. 2013). Therefore, health and ecological risk caused by ANTX producers should be seriously considered.

We hypothesised that in a highly eutrophicated lake with heavy and perennial blooms of microcystin-producing *Planktothrix agardhii*, mass development of ANTX-producing cyanobacteria may also occur under specific water temperature and ratio of easy available forms of nutrients. ANTX production may depend on the same factors which regulate the development of particular *Nostocales* species.

## Materials and Methods

### Study Area

The study was carried out in a small (6 ha), shallow (2.9 m), flow-through and hypertrophic Lake Syczyńskie located in Eastern Poland (51° 17′ N, 23° 14′ E; Ferencz et al. 2014). Agricultural catchment and lack of a sewage system in a village located near the lake were the main reasons of the high trophic status of the lake. Therefore, water blooms caused by filamentous cyanobacteria (*Oscillatoriales*), especially microcystin-producing *P. agardhii* (Pawlik-Skowrońska et al. 2008), have been observed there for many years. The study on the development and toxin production by *P. agardhii* were carried out at the same period as the investigations of *Nostocales* cyanobacteria, and the results were published previously (Toporowska and Pawlik-Skowrońska 2014).

### Field Methods

Water samples for analyses of chemical parameters, phytoplankton (including cyanobacteria) abundance and biomass as well as cyanotoxin detection were taken from February or March to November from 2006 to 2009, from the uppermost (0–0.5 m) water layer in the central part of the lake. The basic physical parameters of the lake water, including temperature, transparency (SD), pH, conductivity and oxygen concentration, were measured in situ. Samples of cyanobacterial scum were also collected from the surface layer of the lake water in both 2006 and 2008.

### Laboratory Methods of Identification and Enumeration of Phytoplankton

The taxonomic identification of phytoplankton (including cyanobacteria) was carried out by light microscopy. *Nostocales* identification and systematics were based mainly on Komárek (1996) and Wacklin et al. (2009), respectively. The quantitative structure of the algal community was analysed by means of an inverted microscope (Utermöhl 1958). For all *Oscillatoriales* and *Nostocales* with straight filaments, 100 μm was set as one individual. One colony of *Dolichospermum* spp. and of coccoid cyanobacteria were recognised as individuals. Phytoplankton biomass was estimated by cell volume measuring (Hillebrand et al. 1999). The species similarities of *Nostocales* assemblages that occurred in the lake were compared using the Jaccard’s index (Jaccard 1912). The index varies from 0 to 1 and was calculated from the following formula:

$$ Sj=\left(a + b + c\right)/\mathrm{a} $$

where

*a*:  = number of species in sample A and sample B (joint occurrence),
*b*:  = number of species in sample B but not in sample A,
*c*:  = number of species in sample A but not in sample B.

### Laboratory Analyses of Water Chemistry and Anatoxin-a Concentration

N-NH_4_, N-NO_3_ and P-PO_4_ concentrations in the lake water were determined according to Hermanowicz et al. (1999), whereas chlorophyll-a concentration according to PN-ISO, 10260 (2002). The Trophic State Index based on water transparency (TSI_SD_) was calculated according to Carlson (1977). The mass ratio of dissolved inorganic nitrogen (DIN) to dissolved inorganic phosphorus (DIP) was calculated.

The extraction of ANTX from cyanobacterial biomass collected on filters GF/C (Whatman) was carried out in 75 % (*v*/*v*) methanol (Merck, pure *p.a.*) acidified with 0.01 M HCl, using ultrasonication (two times for 5 min, 50 W; SONOPULS ultrasonic homogeniser, Bandelin). After centrifugation (14,000 rpm for 10 min at 17 °C) and collection of supernatants, filters with the biomass were back-extracted (once for 5 min), and after centrifugation, the combined supernatants (4.0–4.7 mL) were collected in glass tubes and kept at −20 °C until the day of cyanotoxin analysis. The extraction of ANTX from the 1mL scum of *Dolichospermum lemmermannii*, *Dolichospermum flos-aquae* and *P. agardhii* was carried out in 2 mL of 100 % methanol acidified with 0.01 M HCl using ultrasonication (three times for 5 min). After centrifugation, supernatants were collected and kept at −20 °C. Filtered lake water was evaporated to dry. The residue was extracted in 75 % methanol acidified with 0.01 M HCl. The fluorometric derivatisation of samples was performed as follows: a sample was mixed with 0.1 Na_2_B_4_O_7_ and 4-fluoro-7-nitrobenzofurazan (NBD-F; Fluka) and incubated for 10 min in the dark at room temperature. The reaction was stopped with 1 M HCl. Samples (20 μL) were analysed by means of HPLC with fluorescence detection (Shimadzu; detector parameters ex 470 nm, em 530 nm) according to James et al. (1998). ANTX identification was carried out based on a standard retention time. The calibration curve for quantitative analysis was made with standard ANTX (Tocris, BioScience).

### Statistical Analyses

An indirect multivariate method (DCA) was used to measure and illustrate gradients indicated by *Nostocales* community. Because the length of the gradient was between three and four standard deviations, redundancy analysis (RDA) was used to explore the relationships between the abundance of particular *Nostocales* species, *P. agardhii* and eight environmental variables (water temperature, conductivity, pH, transparency, N-NO_3_, N-NH_4_, P-PO_4_ and DIN/DIP ratio). The data were log-transformed to normalise them. A Monte Carlo analysis with 499 permutations was used to determine the most important variables. Variables which were significant (*P* < 0.05) and influenced cyanobacterial development were bolded. The ordination analyses were performed by means of CANOCO 4.5 software for Windows (ter Braak and Smilauer 2002). Correlation coefficient was also used to study relationships between the biomass of *Nostocales* and *P. agardhii* and physicochemical parameters of lake water, between particular cyanobacteria and between cyanobacteria biomass and concentrations of intracellular ANTX and ANTX and physicochemical parameters of the lake water.

## Results

### Composition of *Nostocales* Community on the Background of Environmental Factors

**Fig. 1:**
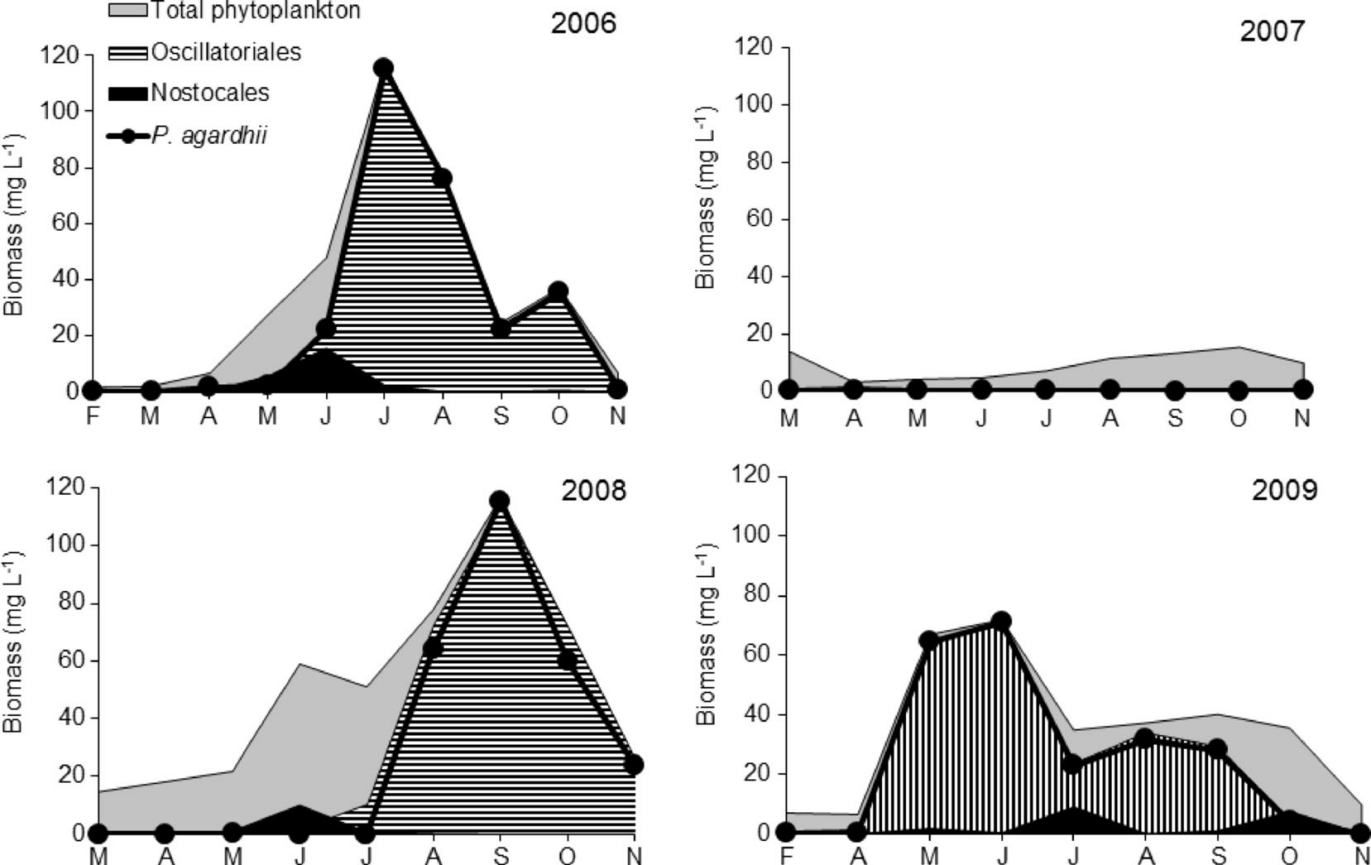
Changes in biomass of *Nostocales* in comparison with the total biomass of phytoplankton and *Oscillatoriales* in Lake Syczyńskie in 2006–2009

**Fig. 2:**
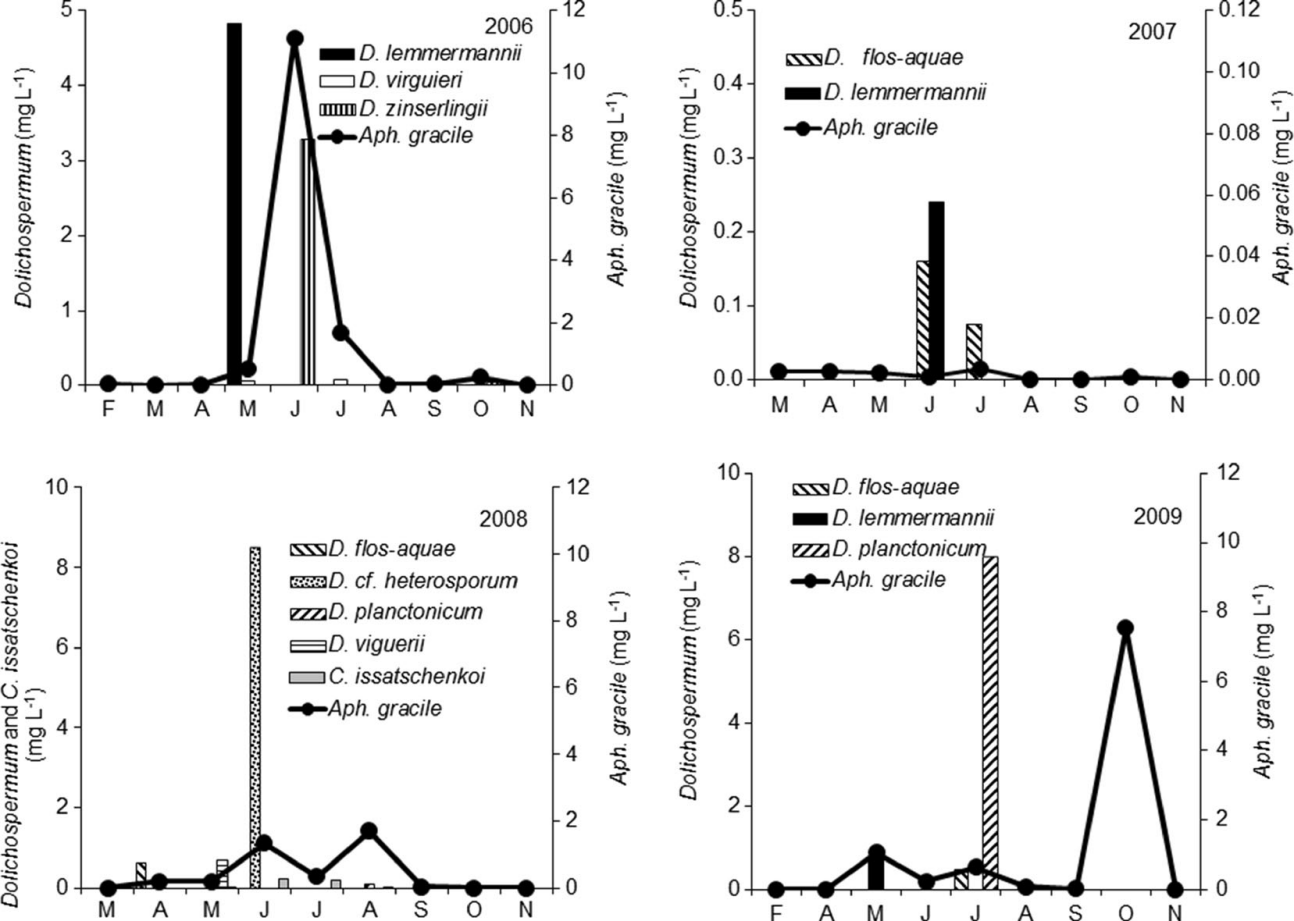
The biomass of the most abundant species of *Nostocales* developed in Lake Syczyńskie in 2006–2009

In the shallow, highly eutrophic (maximum TSI_SD_ = 83), alkaline lake, the physicochemical conditions (Table 1) enhanced the periodical mass development of several species of filamentous cyanobacteria (Figs. 1 and 2 and Table 2) belonging to *Nostocales* species and strong long-lasting water blooms of *P. agardhii* (*Oscillatoriales*). From 5 to 11 taxa of *Nostocales*, occurring in different proportions, were distinguished every year (Table 2). The most frequent species found were *Aphanizomenon gracile*, *D. flos-aquae*, *D. lemmermannii*, *Dolichospermum viguieri*, *Dolichospermum planctonicum* and *C. issatschenkoi* (Fig. 2 and Table 2). The highest species similarity of *Nostocales*, evaluated with the application of the Jaccard index, was found for the years 2006 and 2009 and the lowest for 2007 and 2008 (Table 3).

**Table 1:** Physicochemical characteristics of Lake Syczyńskie water

Years and periods	2006		2007		2008		2009	
	My–Jy	Jy–N	M–A	A–Jy	A–Ag	Ag–N	My–S	My–Jy; O^a^
Parameters	Nost.	Osc.	Osc.	Nost.	Nost.	Osc.	Osc.	Nost.
Water temperature (°C)	22.6	17.3	10.3	20.2	20.3	11.7	18.3	17.5
	(17.6–26.8)	(3.9–25.5)	(7.1–18.4)	(13.4–24.9)	(14.5–22.4)	(6.7–20.0)	(8.4–25.4)	(8.4–25.4)
pH	8.4	7.7	7.8	7.6	8.1	7.8	8.4	8.3
	(8.2–8.6)	(7.4–8.2)	(7.4–8.2)	(7.1–8.2)	(7.7–8.4)	(7.5–8.0)	(8.0–8.9)	(7.7–8.9)
Conductivity (μS cm^−1^)	386	407	603	500	483	403	446	445
	(272–470)	(272–507)	(599–606)	(394–599)	(331–560)	(357–438)	(411–501)	(411–501)
Transparency (SD; m)	0.53	0.30	1.26	1.53	0.52	0.31	0.36	0.42
	(0.17–1.10)	(0.17–0.57)	(0.62–1.89)	(1.20–1.89)	(0.40–0.74)	(0.20–0.40)	(0.25–0.55)	(0.25–0.60)
Dissolved oxygen (mg L^−1^)	14.6	9.5	13.2	8.9	11.4	13.0	13.6	13.7
	(12.0–18.2)	(5.8–12.0)	(10.5–16.0)	(6.8–10.5)	(10.4–12.5)	(10.5–16.2)	(9.4–20.8)	(9.7–20.8)
N-NH_4_ (mg L^−1^)	0.246	0.531	0.160	0.179	0.262	0.515	0.618	0.543
	(0.230–0.263)	(0.263–1.201)	(0.106–0.187)	(0.045–0.247)	(0.125–0.571)	(0.187–0.854)	(0.437–0.800)	(0.147–0.725)
N-NO_3_ (mg L^−1^)	0.089	0.127	0.096	0.096	0.047	0.146	0.089	0.221
	(0.035–0.125)	(0.035–0.260)	(0.003–0.189)	(0.003–0.246)	(0.030–0.064)	(0.097–0.196)	(0.024–0.254)	(0.024–0.884)
P-PO_4_ (mg L^−1^)	0.029	0.105	0.227	0.190	0.022	0.120	0.086	0.081
	(0.017–0.038)	(0.017–0.307)	(0.196–0.257)	(0.050–0.276)	(0.003–0.052)	(0.052–0.240)	(0.012–0.138)	(0.012–0.138)
DIN/DIP ratio	11.8	6.3	1.1	1.5	12.9	4.9	8.2	14.1
	(9.7–17.7)	(4.3–17.7)	(0.7–1.5)	(0.7–1.9)	(3.6–70.1)	(3.6–8.0)	(5.4–57.2)	(4.5–57.2)
Chlorophyll a (μg L^−1^)	104.9	126.2	29.7	13.5	141.9	199.7	82.2	77.9
	(15.4–231.1)	(32.5–325.9)	(8.2–51.2)	(8.2–25.1)	(44.9–256.9)	(66.3–297.9)	(47.5–116.9)	(47.5–116.9)
TSI_SD_	76	77	62	54	69	78	75	74
	(66–73)	(68–84)	(51–67)	(51–57)	(64–73)	(73–83)	(67–80)	(67–80)

Mean and range of values in periods of mass development of *Nostocales* (Nost.) and *Oscillatoriales* (Osc.), *n* = 2–10

*M* March, *A* April, *My* May, *Jy* July, *Ag* August, *S* September, *O* October, *N* November

^a^In October 2009, only *A. gracile* developed massively

**Table 2:** Variability in abundance (range of values) of particular *Nostocales* taxa present in Lake Syczyńskie in the periods of ANTX occurrence in lake water (except 2007, when ANTX was not detected)

Taxa	Abundance (ind. 10^4^ L^−1^ of water)			
	2006 (My–Ag)	2007 (M–O)	2008 (A–Ag)	2009 (My–Jy)
*Aphanizomenon gracile*	0.029–959.4	0–0.3	28.7–116.0	20.0–92.2
*Cuspidothrix issatschenkoi* ^a^	0–6.1	–	0–30.7	0–3.1
*Dolichospermum* cf. *heterosporum*	–	–	0–883.6	–
*D. compactum*	0–0.2	–	0–51.4	0–001
*D. crassum*	–	–	–	0–0.3
*D. flos-aquae* ^a^	0–0.3	0–6.0	0–23.5	0–11.0
*D. heterosporum*	–	–	0–0.001	–
*D. lemmermannii* ^a^	0–181.1	0–9.0	0–0.1	0–15.0
*D. mendotae*	–	–	0–0.001	–
*D. planctonicum*	–	–	0–0.8	0–105.7
*D. viguieri*	0–18.9	0–0.2	0–16.6	0–0.09
*D. zinserlingii*	0–250.4	–	0–12.4	–
*Dolichospermum* sp.	0–0.6	0–0.2	–	0–0.2
Number of taxa	8	5	11	9

*M* March, *A* April, *My* May, *Jy* July, *Ag* August, *O* October; – taxon not found

^a^Main ANTX producers

**Table 3:** Jaccard index of similarity of *Nostocales* assemblages in Lake Syczyńskie in 2006–2009

Year	2007	2008	2009
2006	0.63	0.58	0.78
2007	–	0.33	0.63
2008	–	–	0.58

**Fig. 3:**
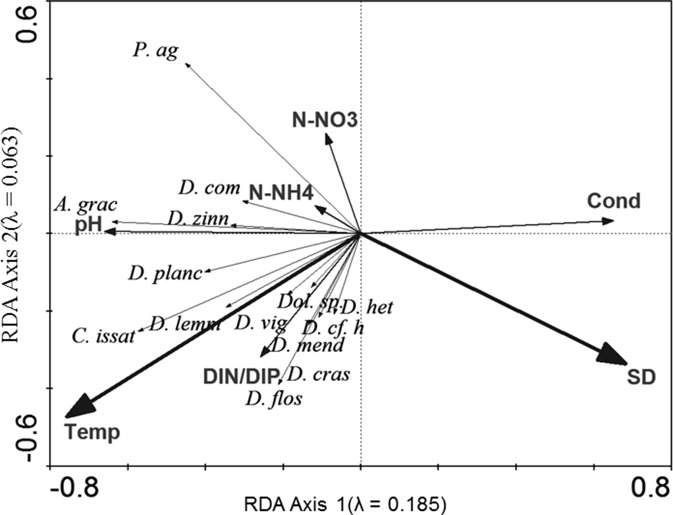
Redundancy analysis (RDA) biplots for *Nostocales*, *P. agardhii* and environmental parameters. The *bolded arrows* indicate the significant variables which had influence on development of cyanobacteria and were based on Monte Carlo permutation test (*P* < 0.05). *Temp* temperature, *SD* transparency, *Cond* conductivity, *N-NH*_*4*_ ammonium nitrogen, *N-NO*_*3*_ nitrate nitrogen, *DIN/DIP* the mass DIN/DIP ratio, *A. grac A. gracile*, *C. issat C. issatschenkoi*, *D. flos D. flos-aquae*, *D. lemm D. lemmermannii*, *D. het D. heterosporum*, *D.* cf. h *D.* cf*. heterosporum*, *D. planc D. planctonicum*, *D. vig D. viguieri*, *D. com D. compactum*, *Dol. sp. Dolichospermum sp.* and *P. ag P. agardhii*

The concentrations of N-NH_4_, N-NO_3_ and P-PO_4_ in Lake Syczyńskie water were generally very high throughout the study period (Table 1). The phytoplankton reached high total biomass with maximum values reaching from 13.73 to 117.30 mg L^−1^ (Fig. 1). It resulted in high-oxygen saturation of water and its very low transparency 0.17–0.20 m (Table 1). The contribution of cyanobacteria in the total biomass of phytoplankton varied considerably and ranged from 7.2 to 99.9 % in 2006, from 0.4 to 8.7 % in 2007, from 0.07 to 99.6 % in 2008 and from 2.5 to 99.2 % in 2009. The total biomass of *Nostocales* reached up to 16 mg L^−1^, and their maximum contribution in the total biomass of cyanobacteria over the study period ranged from 33 to 85 % (Fig. 1). Periodically (Table 2 and Fig. 2), several *Dolichospermum* species achieved high density and biomass. *Dolichospermum* cf. *heterosporum*, *D. planctonicum* and *A. gracile* developed very intensively achieving maximum biomasses equal to 8.50, 8.0 and 11.11 mg L^−1^, respectively, whereas *C. issatschenkoi* reached a much lower biomass with a maximum value equal to 0.22 mg L^−1^ in 2008 (Fig. 2). These diazotrophic *Nostocales* developed in the lake together with *Oscillatoriales* predominated by *P. agardhii*, whose contribution in the total cyanobacterial biomass reached up to 99.2 %. However, the most intensive growth of *Nostocales* was observed before or after the maximum development of *P. agardhii* (Fig. 1) – mainly in spring/early summer seasons or in autumn and at seasonal mean water temperatures higher (17.5–22.6 °C) than those supporting the development of *Oscillatoriales* (10.3–18.3 °C). The development of *Nostocales* was more intense than that of *P. agardhii* at higher ratio (11.8–14.1) of DIN/DIP (Table 1 and Fig. 3). In 2007, a strong decrease in the ratio of DIN/DIP (1.1–1.5), which was due to the decrease in N-NH_4_ and increase in P-PO_4_ concentrations, –essentially decreased both the species richness of *Nostocales* and the total abundance and biomass of cyanobacteria (Figs. 1 and 2 and Table 2). During that time, the *Nostocales* biomass was found to be approximately 20-fold and the *P. agardhii* biomass almost 800-fold lower compared to other years; therefore, the values of TSI_SD_ index decreased from 77 in 2006 to 54 in 2007 but later increased again (Table 1).

The relationships between the abundance of particular *Nostocales* species, *P. agardhii* and environmental variables were also analysed by using RDA analysis (Fig. 3). Monte Carlo permutation test (*P* < 0.05) showed that the most significant factors were water temperature (*λ* = 0.12; *F* = 4.94; *P* = 0.002) and water transparency (*λ* = 0.07; *F* = 2.97; *P* = 0.004). The analysis confirmed that the higher water temperatures were more important for the development of *Nostocales* than that of *P. agardhii*. Water transparency was a consequence of phytoplankton (mostly *P. agardhii*; Table 4) blooms, and this parameter showed much lower values (0.17–1.10 m) in 2006, 2008 and 2009 compared to 2007 (Table 1), when cyanobacterial biomass was low. Transparency was much lower during blooms of *P. agardhii* than during mass development of *Nostocales*. Statistical analysis suggests that this physical factor had no influence on the development of most *Dolichospermum* species but negatively correlated with the growth of *A. gracile*, *D. compactum*, *D. planctonicum* and *D. zinserlinglii*. Interestingly, RDA analysis (Fig. 3) revealed that the DIN/DIP ratio supported the development of most *Dolichospermum* species and *C. issatschenkoi*, and it was of higher importance (*λ* = 0.04; *F* = 1.76; *P* = 0.072) than the concentrations of particular nutrients such as N-NH_4_ (*λ* = 0.03; *F* = 1.22; *P* = 0.316), N-NO_3_ (*λ* = 0.02; *F* = 0.97; *P* = 0.316) and P-PO_4_. Similar results were obtained by the calculation of correlation coefficient (Table 4). However, we observed that water temperature was more important for the growth of *Dolichospermum* spp., whereas the DIN/DIP ratio was found to be more important for the development of *C. issatschenkoi*. Statistical analyses (Fig. 3 and Table 4) confirmed that species belonging to *Dolichospermum* genera and *C. issatschenkoi* reached the highest biomasses during the same periods and at similar environmental conditions. Correlation between their biomasses was equal to 0.49. *A. gracile* growth was favoured in different seasons.

**Table 4:** Correlations between biomass of cyanobacteria and physicochemical parameters of lake water, between particular cyanobacteria, cyanobacteria biomass and concentration of intracellular ANTX and intracellular ANTX and physicochemical parameters of lake waters

	*P. agardhii*	*Dolich.* spp.	*A. gracile*	*C. issatsch.*	ANTX
Water temperature	0.18	0.42*	0.19	0.28	0.34*
pH	0.22	0.22	0.23	0.15	0.29
Conductivity	−0.52*	−0.30	−0.27	−0.38*	−0.39*
Transparency (SD)	−0.60*	−0.16	−0.22	−0.19	−0.26
N-NH_4_	0.20	−0.02	−0.14	0.01	−0.10
N-NO_3_	−0.09	−0.20	0.31	−0.13	−0.14
P-PO_4_	−0.11	−0.14	−0.18	−0.24	−0.34*
DIN/DIP ratio	−0.05	0.29	0.02	0.61*	0.39*
*P. agardhii*	–	−0.08	0.06	−0.12	0.07
*Dolichospermum* spp.	–	–	0.17	0.49*	0.37*
*A. gracile*	–	–	–	0.07	−0.06
*C. issatschenkoi*	–	–	–	–	0.64*

*Dolich.* spp. *Dolichospermum* spp., *C. issatsch. C. issatschenkoi*

*Statistically significant values (*P* < 0.05)

### Anatoxin-a Production in the Lake

**Fig. 4:**
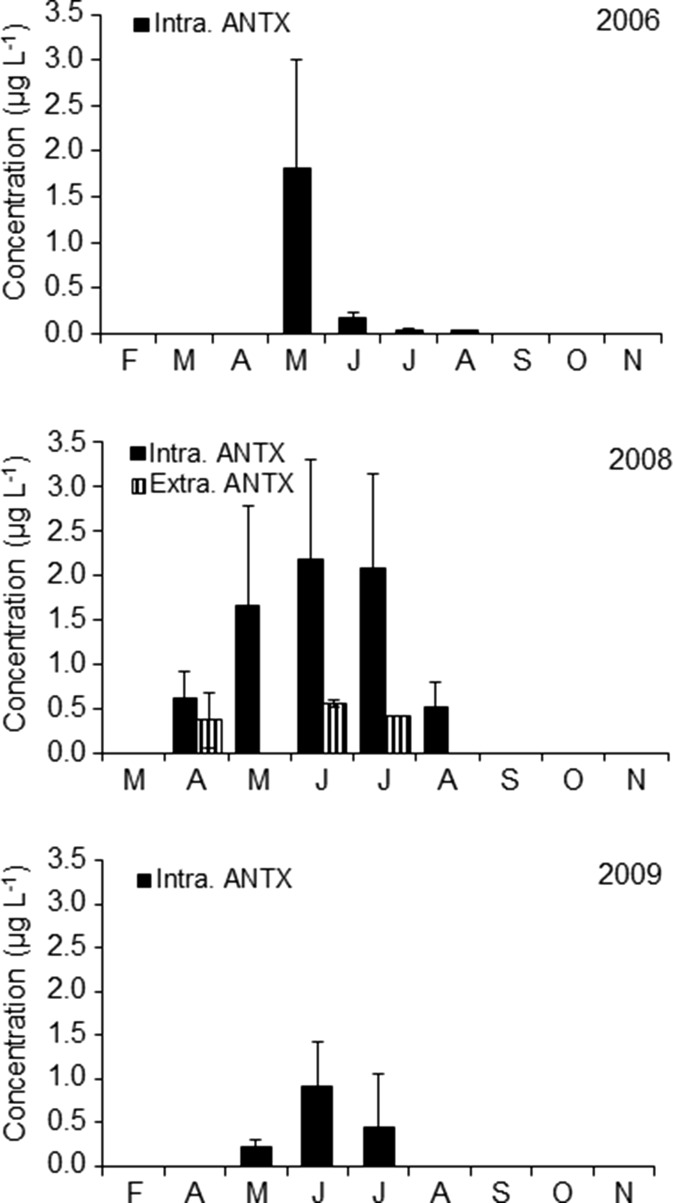
Concentrations of intracellular (intra.) and extracellular (extra.) fraction of anatoxin-a in the water of Lake Syczyńskie in 2006–2009 (mean ± SD)

**Fig. 5:**
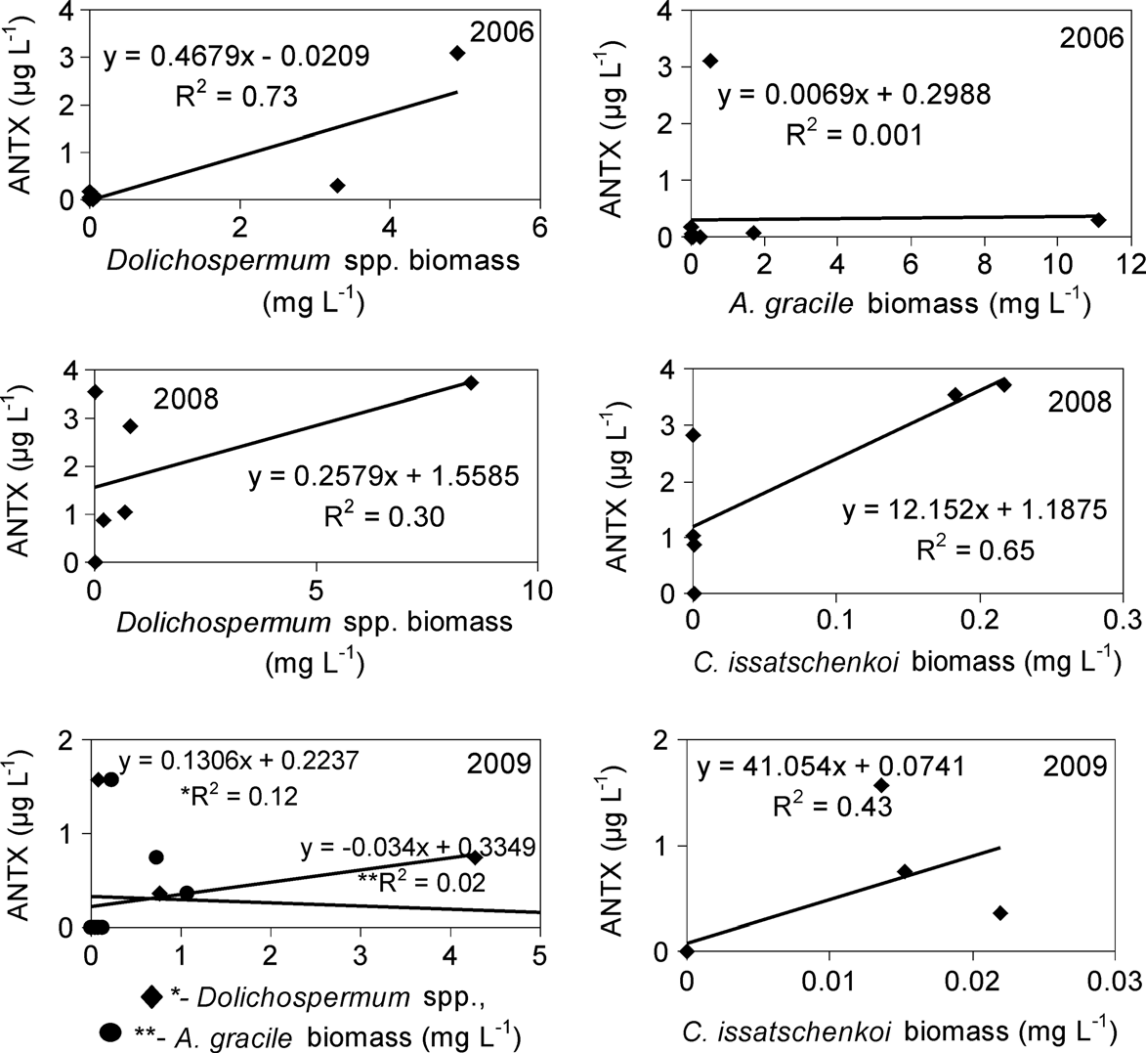
Relationships between the biomass (mg L^−1^) of particular species of *Nostocales* and intracellular anatoxin-a concentrations (μg L^−1^) in water of Lake Syczyńskie

ANTX concentrations in the lake varied considerably within the study years, seasons and months (Fig. 4); however, toxin was detected only during mass development of *Nostocales* (Figs. 1 and 2), and it was not found in the scum of *P. agardhii*. In 2006, the highest concentration of intracellular ANTX in the lake water was equal to 1.82 μg L^−1^ and was observed during *D. lemmermannii* bloom (Fig. 4). Moreover, as much as 3457 μg of ANTX was detected in 1 L of the very dense surface scum created by this species. In 2008 (Fig. 4), from 1.66 to 2.19 μg L^−1^ of intracellular ANTX was found from May to July during the mass development of several *Dolichospermum* species and *C. issatschenkoi*. During that year in spring, 228 μg L^−1^ of intracellular ANTX was also found in the scum created by *D. flos-aquae*. In 2009, concentrations of intracellular ANTX reached from 0.21 to 0.92 μg L^−1^, during which time *D. lemmermannii*, *D. planctonicum* and *D. flos-aquae* were found abundant in the lake except *C. issatschenkoi*. Extracellular ANTX was detected at concentrations from 0.41 to 0.55 μg L^−1^ only in 2008, during which period *Dolichospermum* spp*.* and *C. issatschenkoi* proliferated. Statistical analyses showed that concentrations of intracellular ANTX positively correlated with the biomass of *Dolichospermum* spp. and *C. issatschenkoi* (Table 4). More detailed analysis (Fig. 5) showed that in 2006, intracellular ANTX positively correlated (*R*^2^ = 0.73) with the biomass of all *Dolichospermum* species (Fig. 5), whereas in 2008 and 2009, correlation of cell-bound ANTX with *C. issatschenkoi* biomass (*R*^2^ = 0.65 and 0.43, respectively) was found. However, *Dolichospemum* spp. could also produce ANTX, particularly in 2008 (Fig. 5). No correlation between *A. gracile* biomass and ANTX was found.

**Fig. 6:**
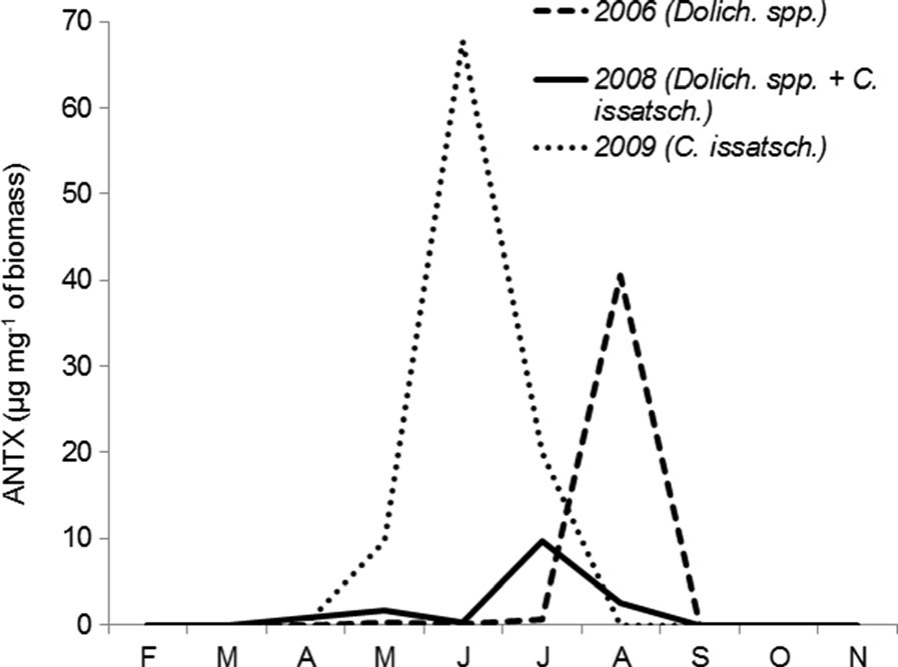
Concentrations of intracellular ANTX per biomass of potential ANTX producers in particular years (*Dolich. Dolichospermum* species, *C. issatsch. C. issatschenkoi*)

It seems that the production of intracellular ANTX was related to the same factors which controlled the development of particular *Nostocales* cyanobacteria (Table 4). ANTX production by *Dolichospermum* spp. was related mainly to water temperature, whereas that by *C. issatschenkoi* to the increasing DIN/DIP ratio and decreasing water conductivity. However, decreasing P-PO_4_ concentrations seemed to be also important. In general, amongst all years, intracellular ANTX was found to be most intensively produced during summer periods, at which time maximum development of particular *Nostocales* was observed and *P. agardhii* was found to be abundantly high or increasing. Intracellular ANTX reached up to 40.6 μg mg^−1^ of the biomass of *Dolichospermum* spp. in 2006, 9.7 μg mg^−1^ of the biomass of *Dolichospermum* spp. and *C. issatschenkoi* in 2008 and 64.3 μg mg^−1^ of the *C. issatschenkoi* biomass in 2009 (Fig. 6).

## Discussion

Ecology of cyanobacteria and cyanobacterial blooms and their causes and effects attract more and more attention for mass development of neurotoxin producers (Sivonen et al. 1990; Henriksen et al. 1997; Bumke-Vogt et al. 1999; Pawlik-Skowrońska et al. 2004; Pawlik-Skowrońska et al. 2013; Heath et al. 2016; Cirés and Ballot 2016). This 4-year study carried out in the shallow hypertrophic lake dominated by *P. agardhii* (Pawlik-Skowrońska et al. 2008; Toporowska et al. 2014) showed a high variability both in qualitative and quantitative structure of assemblages of ANTX-producing *Nostocales. Nostocales* developed massively in spring and at early summer seasons at higher water temperatures compared to *Oscillatoriales*. The temperatures were similar to the optimum temperatures (20–25 °C) reported by Rapala et al. (1993) and were higher than those characteristic for *Oscillatoriales* (Dokulil and Teubner 2000). However, *Nostocales* proliferated also in other seasons at temperatures from 13.2 to 28.8 °C (Bumke-Vogt et al. 1999; Pawlik-Skowrońska et al. 2004). Therefore, other factors such as fluctuations in concentration and/or proportions of nutrients seem to be more important for the growth of *Nostocales*. Majority of reports (Rapala et al. 1993; Catherine et al. 2008; Altman and Pearl 2012), however, showed the effect of individual nutrient compounds. In the lake studied, the DIN/DIP ratio, not concentrations of particular dissolved nutrients, was next to the temperature and water transparency, which was related to *P. agardhii* biomass, the most important factor that modulated the proliferation of *Nostocales* cyanobacteria. At a very low DIN/DIP ratio of 0.7–1.9, a decrease in the abundance and biomass of both *Nostocales* and *Oscillatoriales* resulted in the elimination of cyanobacterial blooms, despite optimal water temperatures and high-nutrient concentrations in the lake water. This suggests that besides increasing the total N/P ratio over 20, providing eukaryotic algae as competitors over cyanobacteria (Pearl 2008), decreasing DIN/DIP ratio to a very low level (ca. 1/1) may also be one of the methods of controlling cyanobacterial blooms. A long-term study carried out in Zemborzycki Reservoir showed that cyanobacteria biomass decreased with decreasing P-PO_4_ concentrations and with increasing DIN/DIP ratio (above 23) (Pawlik-Skowrońska et al. 2013). A decrease in *Nostocales* biomass with increasing biomass of *P. agardhi*i, decreasing water transparency and increasing shading was typical for both Lake Syczyńskie and Zemborzycki Reservoir. The decrease in light had a strong role in the decline of *Dolichospermum circinalis* bloom in hypertrophic reservoir in Argentina (Fernández et al. 2015). No data exist on the potential toxic effect of *P. agardhii* on ANTX producers. But, recent experiment by Ma et al. (2015) showed that different species of *Microcystis* inhibited the development of *A. flos-aquae* to various degrees; however, pure microcystin-LR did not inhibit *A. flos-aquae* development. This suggests some possible allelopathic interactions between cyanobacteria, but further studies in this field are required.

Mass development of *Nostocales* was observed in other shallow and eutrophic lakes in Europe (Bumke-Vogt et al. 1999; Pawlik-Skowrońska et al. 2004; Ballot et al. 2010) or South America (Becker et al. 2004; Ruiz et al. 2013), and other factors controlling *Nostocales* growth and structure were revealed. For example, in a shallow subtropical Brazilian lake, in which the abundance of *D. circinalis* and *D. spiroides* reached up to 4.5 × 10^6^ ind. L^−1^ (Becker et al. 2004), the maximum bloom was preceded by an event of strong turbulence in the lake system. This resulted in the availability of nutrients and dispersion of the cyanobacteria akinetes, stored in the sediment, into the water column. Later blooms were supported by progressive reduction in wind velocity and longer stabilisation period in the water column. Recently, Molot et al. (2014) suggested that anoxia may play a critical role in bloom formation and the consequent release of ferrous iron from the sediments. In Lake Syczyńskie, anoxic conditions occurred in winter/spring period in 2006 (Toporowska et al. 2010), which could favour later cyanobacterial blooms. Vegetation period in 2007 was free of blooms and was preceded by warm winter preventing anoxia. Simultaneously, an increase in supply dynamics, such as runoff, hypodermic and underground supply, favoured water mixing, as well as a negative balance of total phosphorous, soluble reactive phosphorous and ammonium (Dawidek et al. 2009). As a consequence, changes in nutrient concentrations and ratios in the lake water were observed. Therefore, in the studied lake, complex lake-catchment processes probably play a key role in the formation of cyanobacterial blooms. These processes may be very dynamic (Dawidek et al. 2009; Ferencz et al. 2014) and shape most physicochemical parameters of the lake water, regulating the growth of particular cyanobacterial species.

In Lake Syczyńskie, the development of *Aphanizomenon* species was more stable and long lasting than species belonging to *Dolichospermum* genera. Moreover, *A. gracile* reached a high biomass also in cold periods, even under the ice cover, at a water temperature ≤4 °C (Toporowska et al. 2010). Occurrence of *Aphanizomenon* in temperate lakes seems to be more frequent than that of *Cuspidothrix* (Ballot et al. 2010; Kobos et al. 2013). For example, Ballot et al. (2010), amongst 61 *Aphanizomenon* strains found in a German lake in years 2007–2008, classified 59 as *A. gracile* and only 2 as *C. issatschenkoi*. One of them was a very effective ANTX producer (2.35 mg g^−1^ FW)*.* It seems that *C. issatschenkoi*, *D. flos-aquae* and *D. lemmermannii* were the main ANTX producers in Lake Syczyńskie. The maximal production of ANTX reaching up to 67.7 μg mg^−1^ FW of cyanobacterial biomass was much higher compared to other water bodies. For example, *A. flos-aquae* and *Dolichospermum* spp. from Portuguese freshwaters produced more than 20 μg of ANTX per gram of dry weight (Osswald et al. 2009). Production of ANTX is strain specific, but our field study showed that it may change with changing abiotic factors, which also influence the cyanobacterial growth. This is in agreement with the experiments by Rapala et al. (1993) and Osswald et al. (2009). Our results suggest that water temperature, DIN/DIP ratio, conductivity and P-PO_4_ concentrations had the most significant effect on ANTX production. According to Rapala et al. (1993), the optimal temperature for ANTX production was between 19.8 and 22 °C. But, decrease in toxin levels at higher temperatures seemed to depend on the species since the growth of both toxic *Anabaena* (now *Dolichospermum*) cultures suffered, whereas *Aphanizomenon* grew best at 30 °C. Also, high intensity of light limited ANTX production in *Dolichospermum* but not in *Aphanizomenon*. Light limitation caused by an increased in the abundance of *P. agardhii* might intensify ANTX production in Lake Syczyńskie. Lowest concentrations of ANTX in *Nostocales* biomass found in periods of their most intensive mass development may also support the growth-differentiation balance hypothesis (Herms and Mattson 1992), suggesting that actively dividing and expanding cells are less likely to produce secondary metabolites. We did not find correlations between ANTX production and ammonium and nitrate nitrogen, which is contrast to the findings of Rapala et al. (1993), who showed that particularly N_2_ or N-NH_4_ limitation caused an increase in ANTX production. The significant negative correlation observed between ANTX and P-PO_4_ concentrations is in opposition to the findings of Rapala et al. (1993) and in agreement with recent experiment carried out on benthic *Phormidium* sp. by Heath et al. (2016). The experiment suggested that ANTX may be produced as a stress response to growth-limiting conditions because toxin concentrations were found to be the lowest under high-nitrogen and high-phosphorus treatment supporting the above-mentioned growth-differentiation balance hypothesis.

The capability of some *Nostocales* to producing ANTX as well as several different cyanotoxins and other metabolites of still unknown biological activity (Welker and Döhren 2006) increases their threat to aquatic ecosystems. For example, *D. lemmermannii* was confirmed as a producer of neurotoxic anatoxin-a(s), saxitoxins and hepatotoxic microcystins (MCs) (Sivonen et al. 1990; Henriksen et al. 1997; Lepistö et al. 2005), *D. flos-aquae* as a producer of microcystins (Sivonen et al. 1992), which were found also in the scum of *D. flos-aquae* from Lake Syczyńskie (data not published), whereas invasive *C. issatschenkoi* (Kastovsky et al. 2010) as a producer of paralytic shellfish poison (PSP) (Li et al. 2003). *A. gracile* was previously identified as a PSP and cylindrospermopsin producer (Rücker et al. 2007; Kokociński et al. 2013). ANTX production by this species has not been proved neither by our field study nor by previous laboratory and field investigations (Cirés and Ballot 2016). Due to toxin production, even a short-term mass appearance of different *Nostocales* species seems to be a real hazard for freshwater ecosystems and their users. Risk to human health associated with the environmental occurrence of anatoxins was reviewed recently by Testai et al. (2016).

Summing up, in a shallow and highly eutrophicated water body, mass development of *Nostocales*, potential ANTX producers, may depend on water temperature, but it seems to be controlled mainly by DIN/DIP ratio. In contrast to the long-lasting *P. agardhii* blooms, the periodical predominance of particular *Nostocales* genus, especially belonging to *Dolichospermum* genus, may change very quickly. It seems that ANTX production is controlled by more complex factors than the development of particular *Nostocales* species. The impact of development of MC-producing *P. agardhii* cannot be excluded, and experimental studies on *Nostocales*-*Oscillatoriales* interactions are strongly required. Evaluating the impact of varying factors on *Nostocales* community dynamics and ANTX production in eutrophic water bodies seems to be a key to develop strategies to avoid blooms of toxigenic cyanobacteria. There is a strong need of further field and experimental studies. We suggest that due to the global warming and increasing problem of toxigenic cyanobacterial blooms, neurotoxic ANTX should be considered as a pollutant of freshwaters, which needs to be monitored.
